# Joint position and force senses in young female tennis players and untrained adolescents

**DOI:** 10.1371/journal.pone.0312483

**Published:** 2024-10-22

**Authors:** Bartłomiej Niespodziński, Tomasz Waldziński, Aleksandra Durzyńska, Jan Mieszkowski, Małgorzata Knaś, Andrzej Kochanowicz

**Affiliations:** 1 Department of Biological Foundations of Physical Education, Faculty of Health Sciences and Physical Education, Kazimierz Wielki University, Bydgoszcz, Poland; 2 Faculty of Health Sciences, University of Lomza, Lomza, Poland; 3 Department of Gymnastics and Dance, Gdańsk, University of Physical Education and Sport, Gdańsk, Poland; Brunel University London, UNITED KINGDOM OF GREAT BRITAIN AND NORTHERN IRELAND

## Abstract

The aim of the study was to determine the differences between tennis players and untrained peers in the development of upper limb proprioception in 10–15-year-olds. A group of 67 girls (12.75 ± 1.46 years old), including 33 tennis players and 34 age-matched untrained controls, was divided into three age groups: A1, 10–11-years-old; A2, 12–13-years-old; and A3, 14–15-years-old. Joint position sense (JPS) and force sense (FS) were assessed by reproducing memorized target angle or torque value of three joints: glenohumeral, elbow, and radiocarpal. The JPS error for the elbow joint in group A1 was 71% and 80% higher (p < 0.01) than that in groups A2 and A3, respectively, and the performance of all tennis players was 27.5% (p = 0.01) better than that of untrained controls. For FS, proprioception of only the more demanding task tested (reproduction of 50% maximal voluntary contraction) and specific function (elbow and radiocarpal extension, and glenohumeral internal rotation) showed development with age. The error values for elbow extension (A1, A2) and the glenohumeral joint (A3) of tennis players were lower than those of age-matched controls. We conclude that the development of FS in the upper limb varied and was related to the specific functions and joints. The 10–13-year-old tennis players showed elbow extensor FS performance at the level of the older participants, while the 14–15-year-old tennis players were characterized with superior FS internal rotation performance in the glenohumeral joint.

## Introduction

Proprioception is the awareness of the body in space [1]. The sense of proprioception is crucial for the musculoskeletal system, as it allows it to control the movement of the human body in space and the movement of body parts relative to one other. Typically, three modalities of proprioception are recognized, i.e. the sense of balance, kinaesthesia, and the sense of force [2]. While the sense of balance is associated with the vestibular system and is commonly investigated in overall balance tasks [3], the effects of kinaesthesia and force sense (FS) can be observed in particular in the joints and muscles associated with them. Kinaesthesia allows to differentiate joint position and, thus, joint movement, and FS enables differentiation of the magnitude of muscle tension, applied effort, or perceived weight [4]. Each pair of human limbs play a specific role in daily life, wherein the lower limbs are, among others, adapted to locomotion and the upper limbs are adapted to grasping and manipulation. The same applies to individual joints and, consequently, the glenohumeral, elbow, and wrist joints differ in structure and function. Hence, the proprioception capabilities of these joints should also differ. To date, the knowledge of proprioceptive modalities of the upper limb has been mostly limited to kinaesthesia and the elbow joint. Much less is known about FS and other proprioceptive modalities of other joints, such as the wrist joint (radiocarpal) and shoulder joint (glenohumeral).

The development of proprioceptive skills is training-[5] and ontogeny-dependent [6–8]. Therefore, proprioception is of particular importance for children and adolescents practicing sports. On the one hand, high levels of proprioceptive abilities are essential for high sports performance in many sports [9–11]. On the other hand, its insufficiencies increase the risk of injuries, especially in overhead sports [12–15]. Evaluation of differences and/or changes in different age groups of untrained individuals and athletes is one of the methods to get insight into possible training adaptation on the background of typical physical development [16]. This knowledge is important in understanding how specific sport training modifies the proprioception performance, which can be used in early optimization of the process of conditioning targeted for better performance, but also to prevent undesirable changes early enough to reduce the risk of injury.

One sport characterized by a perfect control of upper limb movement is tennis. Specifically, in tennis, players have to operate a racquet with high precision to direct a ball to a desired position, and using adequate strength to give the ball sufficient speed to prevent its receipt by the opponent [17]. Tennis players, through proprioception, have to immediately and precisely adjust their upper limb’s joint position adequate to the ball path anticipation. Moreover, tennis players have to utilize FS to regulate the amount of muscle strength used during strokes so that they are as strong as possible but also land a ball within the court. These tasks are even more difficult because the upper limb joints do not act freely but have an extension in the form of the racquet. The understanding of the development of proprioceptive modalities of specific joints of the upper limb, especially FS, in tennis players, is limited and focused rather on the sense of balance [18]. Some observations [19] point out that at the age of 12, there is a tendency for improvement in shoulder joint position sense (JPS) performance in tennis players. Moreover, it was shown that JPS in hip and knee joints was positively related to tennis experience in adult athletes [20]. Thus, we hypothesize that older children and with higher tennis experience should exhibit better proprioception performance.

Accordingly, the aim of the current study was to evaluate the differences in JPS and FS in the upper limb of untrained girls in three age groups (10–11, 12–13, 14–15), and to compare the proprioception test results with those of experienced tennis players in these age groups.

## Materials and methods

### Experimental approach to the problem

In this cross-sectional study, a group of tennis players and age-match controls underwent proprioception evaluation in the form of FS and JPS testing of the dominant upper limb. All procedures took place at the Laboratory of Physical Effort in Gdańsk University of Physical Education and Sport (Gdańsk, Poland) between 09.11.2020–25.04.2021. Before testing, the participants familiarized themselves with all the procedures.

### Participants

The recruitment of participants started on 2.11.2020 and ended on 16.04.2021. Sixty-seven girls (12.75 ± 1.46 years old) participated in the study, of which 33 were tennis players and 34 were age-matched untrained controls. The participants were divided into three age groups: A1, 10–11-years-old (n = 23); A2, 12–13-years-old (n = 22); and A3, 14–15-years-old (n = 22). The tennis players were selected by coaches from seven regional clubs as the most talented players based on their tennis abilities and competitive performance (measured by rankings). The untrained control group was recruited from a local school (Gdańsk, Poland) and declared no participation in structured sports training or similar. Group characteristics are shown in Table 1. Both untrained controls and tennis players attended obligatory physical education classes 3 × 45 min per week. The study was performed according to the Declaration of Helsinki. Bioethics Committee for Clinical Research of the Regional Medical Society in Gdansk approved the study (KB-25/20). Each participant and their legal guardians gave written informed consent for the study.

**Table 1:** Characteristics of the study participants (mean ± standard deviation).

Variable	Tennis players [age, in years]			Untrained control [age, in years]		
	10–11	12–13	14–15	10–11	12–13	14–15
	(n = 12)	(n = 10)	(n = 11)	(n = 11)	(n = 12)	(n = 11)
**Body mass [kg]**	42.65 ± 7.68	47.52 ± 5.87	52.94 ± 8.23	42.22 ± 10.34	47.78 ± 8.79	59.41 ± 10.04
**Height [cm]**	151.92 ± 9.24	161.1 ± 5.30	164.09 ± 7.13	150.36 ± 7.64	158.5 ± 7.93	163.73 ± 5.46
**Training experience [years]**	4.5 ± 0.5	6.7 ± 0.58	8.1 ± 0.75	–	–	–
**Week training volume [hours]**	6–10	8–12	11–15	–	–	–

### Procedures

#### JPS testing

The procedure was performed using an isokinetic dynamometer Biodex System 4 (Biodex Medical Systems, Inc., Shirley, NY, USA). The participants were seated in an adjustable seat of the device with the backrest adjusted to their body diameters, according to the manufacturer’s guidelines. The position was stabilized using leather straps. JPS was tested for three joints: glenohumeral, elbow, and radiocarpal. For glenohumeral joint testing, the participant’s arm was abducted at 90° in the frontal plane and flexed at 90° at the elbow joint. The hand in neutral position was holding the dynamometer arm and the rotation shaft of the device was aligned with the olecranon. In this position, the external and internal rotation of the glenohumeral joint was tested. During the elbow joint evaluation, the participant’s glenohumeral joint was flexed at 45°, and the tested arm was supported by a pad placed under the olecranon. The hand was oriented in a neutral position and the rotation shaft was aligned with the lateral epicondyle of the humerus. In this position, flexion and extension of the elbow joint were tested. JPS in the radiocarpal joint was assessed in a position wherein the forearm was laid on the support pad in pronate position. The device’s arm was held in such a way that the rotation shaft was aligned with the ulnar styloid. In this position, wrist flexion and extension were evaluated.

During testing, the participants were asked to remember the target joint position of the dominant upper limb set by an investigator, without visual and sound cues. Then, the position was randomly changed and the participant had to indicate the memorized position of the same limb. For each joint, two modes of movement were tested: the active mode, wherein the participant reproduced the target joint angle by moving the dynamometer arm by themselves, and the passive mode, wherein the dynamometer arm moved automatically at a speed of 0.5°/s. In both cases, when the participants thought that they have achieved the target angle, they pushed an indicator button held in the opposite hand. The following target angles were tested: for the glenohumeral joint, 30° of the internal and external rotation; for the elbow joint, 60° and 90° of flexion; and for the radiocarpal joint, 15° of the flexion and extension (dorsiflexion). Participants made three attempts at each target angle and each mode. The order of joint, testing mode, and target angles was randomized.

#### FS testing

The procedure was performed using the same device, and positions and joints as for JPS testing. The testing was performed under isometric conditions. For the glenohumeral joint, the position of the joint was set to 0° (between the external and internal rotation), and the torque of the external and internal rotation was evaluated. The elbow joint was set to 90° of joint flexion, and the flexion and extension were evaluated. For the radiocarpal joint muscles, the wrist was set in neutral position (0°) between flexion and extension, and these two functions were evaluated. The testing was performed by ipsilateral reproduction of the memorized isometric torque level via visual feedback, wherein the participant saw, on a display in front of them, the target level of exerted torque, and then, after a 5 s rest, had to reproduce the same outcome without any visual or sound cues. Before actual testing, the participants performed three maximal voluntary isometric contractions (MVC) for each tested joint and muscle function. The peak value from these three measurements was used as a reference for the target torque level. For each joint and function, the ability to reproduce 20% and 50% MVC was tested. The participants had three attempts at each target torque level and each mode. The order of joint, muscle function, and target torque level testing was randomized.

### Data curation

The JPS outcome was measured in degrees and the FS outcome was measured as percentage points (normalized by MVC). The following JPS and FS outcomes were analysed: absolute error (AE), constant error (CE), and variable error (VE). The AE was calculated as the mean absolute difference between the target value and the reproduced value from all trials. This indicated the magnitude of the error, where results closer to zero represent better performance. The CE was determined as the mean difference between the target value and the reproduced value from all trials. This indicated the direction of the error (undershot by negative values or overshot by positive values). The VE was calculated as the standard deviation of the CE, and represented error repeatability. The reliability of JPS and FS testing using the Biodex Systems has been previously shown to be sufficient, with the intra-class correlation coefficient of 0.78 and 0.81, respectively [21].

### Statistical analysis

All data are shown as the mean ± standard deviation (SD). To assess the difference between tennis players and untrained controls in specific age groups, a set of two-way (2 × 3) analysis of variance (ANOVA) tests was performed. The first factor, *group*, indicated tennis practice, and divided the participants into two groups: tennis players and untrained controls. The second factor, *age*, indicated developmental and training experience differences, and was represented by three age groups: 10–11.9-years-old, 12–13.9-years-old, and 14–15.9-years-old. The assumptions of ANOVA test, including normal distribution (Shapiro—Wilk test) and homogeneity of variance (Levene test), were checked prior to detailed analyses, and no basis to reject them were identified. In the case of a significant effect of factor interaction, Tukey’s post-hoc test was performed. The outliers whose values exceed three standard deviation were excluded from the analysis. In addition, the effect size was estimated by using eta-squared statistics (η^2^), so that values less than or equal to 0.01, 0.06, and 0.14, and >0.14 indicated trivial, small, moderate, and large effect, respectively [22]. The level of significance for all tests was set at α = 0.05. The required sample size of 64 participants was determined using G*Power software ver. 3.1.9.4. (Franz Faul et al., Universität Kiel, Kiel, Germany) for a large effect size and a power of 0.80. All tests were performed using the Statistica 13.3 software (TIBCO Software Inc., Palo Alto, CA, USA).

## Results

### JPS analysis

**Fig 1:**
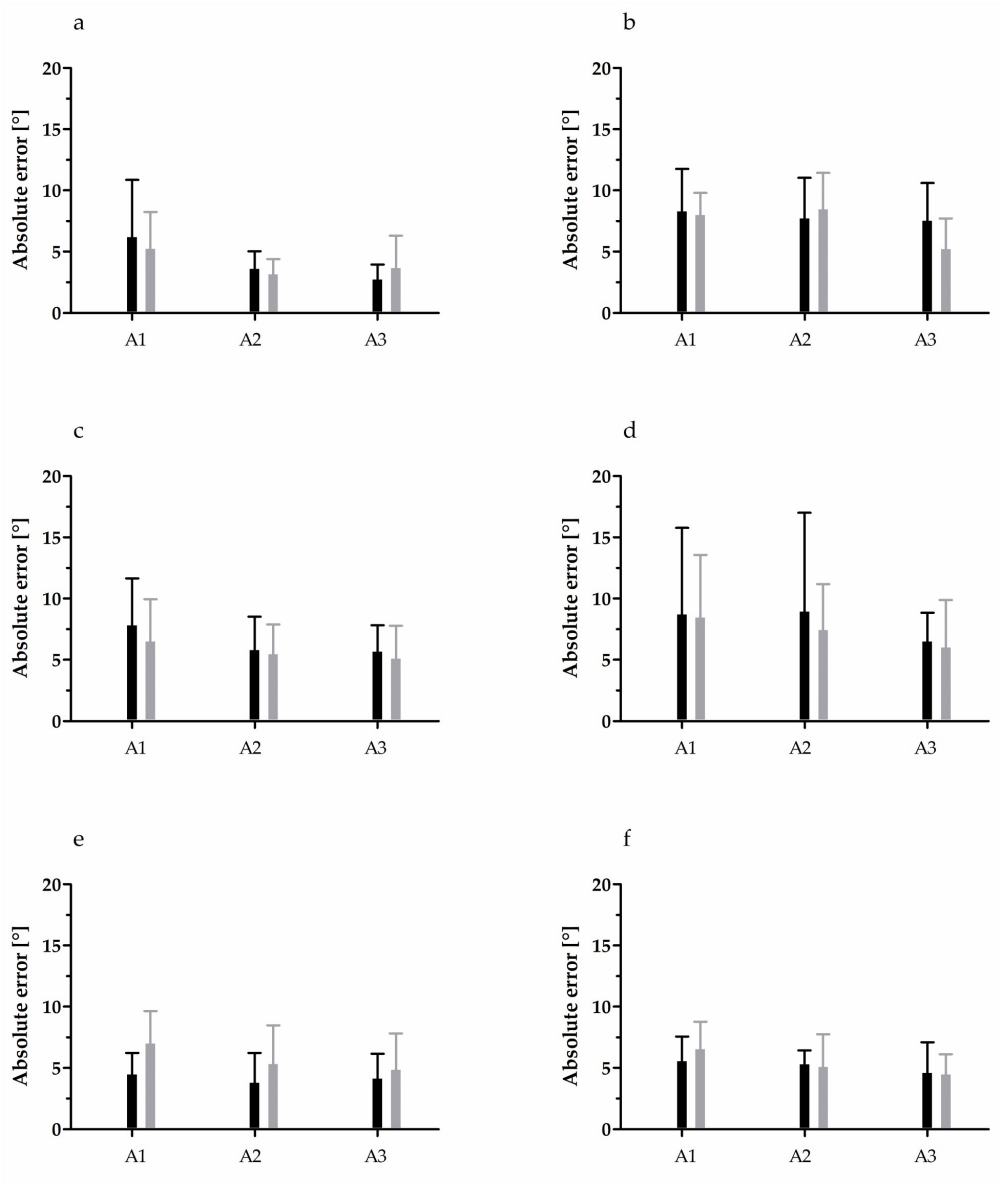
Absolute error in JPS testing. Black bars, tennis players; grey bars, untrained controls. Left-hand panels, active reproduction mode; right-hand panels, passive reproduction mode. (a, b) Elbow joint; (c, d) radiocarpal joint; (e, f) glenohumeral joint. A1, 10–11-year-olds; A2, 12–13-year-olds; A3, 14–15-year-olds. Data are presented as arithmetic mean ± standard deviation.

The results of AE, CE, and VE analyses for JPS are depicted in Fig 1 and Table 2, accordingly. While no interaction between the analysed factors was detected, effects of the main factors were observed. The AE of active reproduction in the glenohumeral joint of tennis players was 27.5% lower than that in the untrained controls (F = 6.55, p = 0.01, η^2^ = 0.1). Further, the VE was highest in the A1 group, regardless of tennis training (F = 4.16, p = 0.02, η^2^ = 0.12). Similar was observed for passive reproduction, but the VE in the A1 group was higher (by 50%) than that only in the A3 group (F = 3.86, p = 0.03, η^2^ = 0.11). In the case of JPS in the elbow joint, a significant effect was only detected for the age factor. In active reproduction test, the AE in the A1 group was 71% and 80% higher than that in the A2 and A3 groups, respectively (F = 6.06, p < 0.01, η^2^ = 0.17), and the VE was 115% and 83% higher in the same comparisons, accordingly (F = 5.99, p < 0.01, η^2^ = 0.16). No differences were observed for the radiocarpal joint.

**Table 2:** Constant (CE) and variable (VE) error in joint position testing (mean ± standard deviation).

Joint		10–11-years-old		12–13-years-old		14–15-years-old	
		Tennis players (n = 12)	Untrained controls (n = 10)	Tennis players (n = 11)	Untrained controls (n = 11)	Tennis players (n = 12)	Untrained controls (n = 11)
Active reproduction (°)							
**Elbow**	**CE**	2.73 ± 4.80	1.59 ± 3.22	0.69 ± 3.10	1.34 ± 2.53	0.05 ± 2.16	1.23 ± 2.64
	**VE**	6.21 ± 4.69	5.71 ± 4.78	2.86 ± 1.08	2.71 ± 1.25	2.44 ± 1.01	4.08 ± 4.05
**Radiocarpal**	**CE**	–0.57 ± 4.17	–1.28 ± 4.26	0.74 ± 4.34	–0.83 ± 2.79	–0.25 ± 2.83	–0.48 ± 4.71
	**VE**	8.23 ± 4.37	6.12 ± 4.18	5.29 ± 3.57	5.69 ± 3.02	5.68 ± 2.43	4.35 ± 2.45
**Glenohumeral**	**CE**	4.8 ± 2.75	2.24 ± 5.58	2.39 ± 2.32	2.15 ± 4.04	0.64 ± 1.94	2.14 ± 5.03
	**VE**	5.62 ± 2.82	5.81 ± 3.15	3.57 ± 2.07	4.28 ± 2.18	4.05 ± 1.99	3.92 ± 1.61
Passive reproduction (°)							
**Elbow**	**CE**	–0.41 ± 5.24	–2.10 ± 2.35	–4.57 ± 2.69	–3.33 ± 5.92	–4.49 ± 5.03	–1.15 ± 2.52
	**VE**	7.86 ± 3.61	8.35 ± 2.09	6.37 ± 4.13	6.84 ± 2.36	5.20 ± 3.16	5.43 ± 2.54
**Radiocarpal**	**CE**	–2.29 ± 7.59	–8.27 ± 5.34	–7.95 ± 9.06	–6.93 ± 4.00	–6.23 ± 2.99	–4.48 ± 5.60
	**VE**	5.79 ± 7.52	2.84 ± 1.95	2.95 ± 1.79	3.38 ± 2.69	3.21 ± 2.00	2.12 ± 1.81
**Glenohumeral**	**CE**	–2.47 ± 3.49	–4.81 ± 2.92	–2.56 ± 3.53	–3.27 ± 4.01	–2.79 ± 4.07	–1.75 ± 3.87
	**VE**	4.58 ± 2.52	4.93 ± 2.51	4.78 ± 1.56	3.50 ± 1.65	2.95 ± 1.25	3.35 ± 1.77

### FS analysis

**Fig 2:**
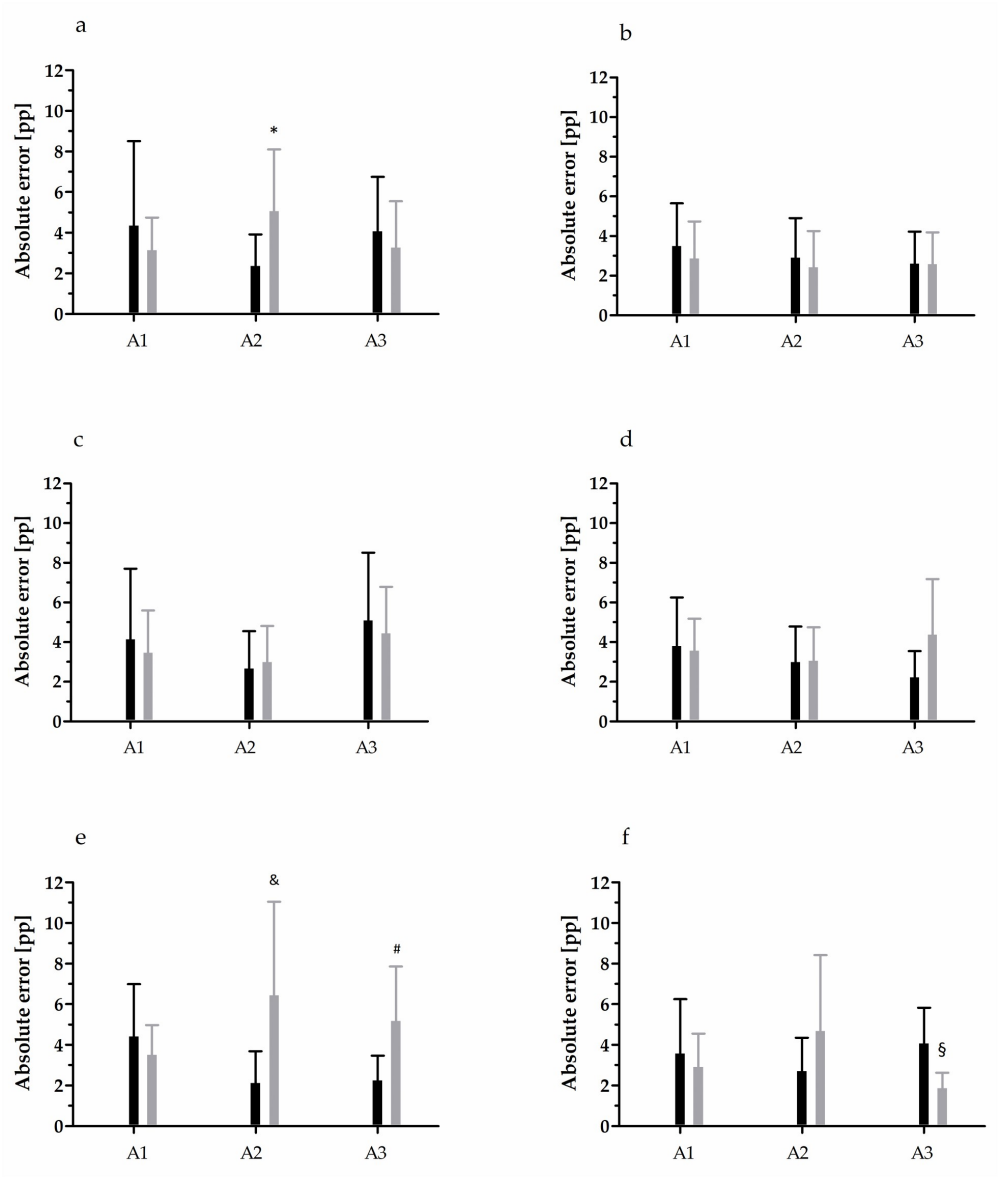
Absolute error in 20% maximal voluntary contraction force sense testing. Black bars, tennis players; grey bars, untrained controls. (a) Elbow extension; (b) elbow flexion; (c) radiocarpal extension; (d) radiocarpal flexion; (e) glenohumeral internal rotation; (f) glenohumeral external rotation. Data are presented as arithmetic mean ± standard deviation. A1, 10–11-year-olds; A2, 12–13-year-olds; A3, 14–15-year-olds. Pp, percentage point. * Significant difference vs. untrained 12–13-year-olds at p < 0.05; ^&^ significant difference vs. untrained 10–11-year-olds, and 12–13- and 14–15-year-old tennis players at p < 0.05; ^#^ significant difference vs. 12–13- and 14–15-year-old tennis players at p < 0.05; ^§^ significant difference vs. 14–15-year-old tennis players and untrained 12–13-year-olds at p < 0.05.

**Fig 3:**
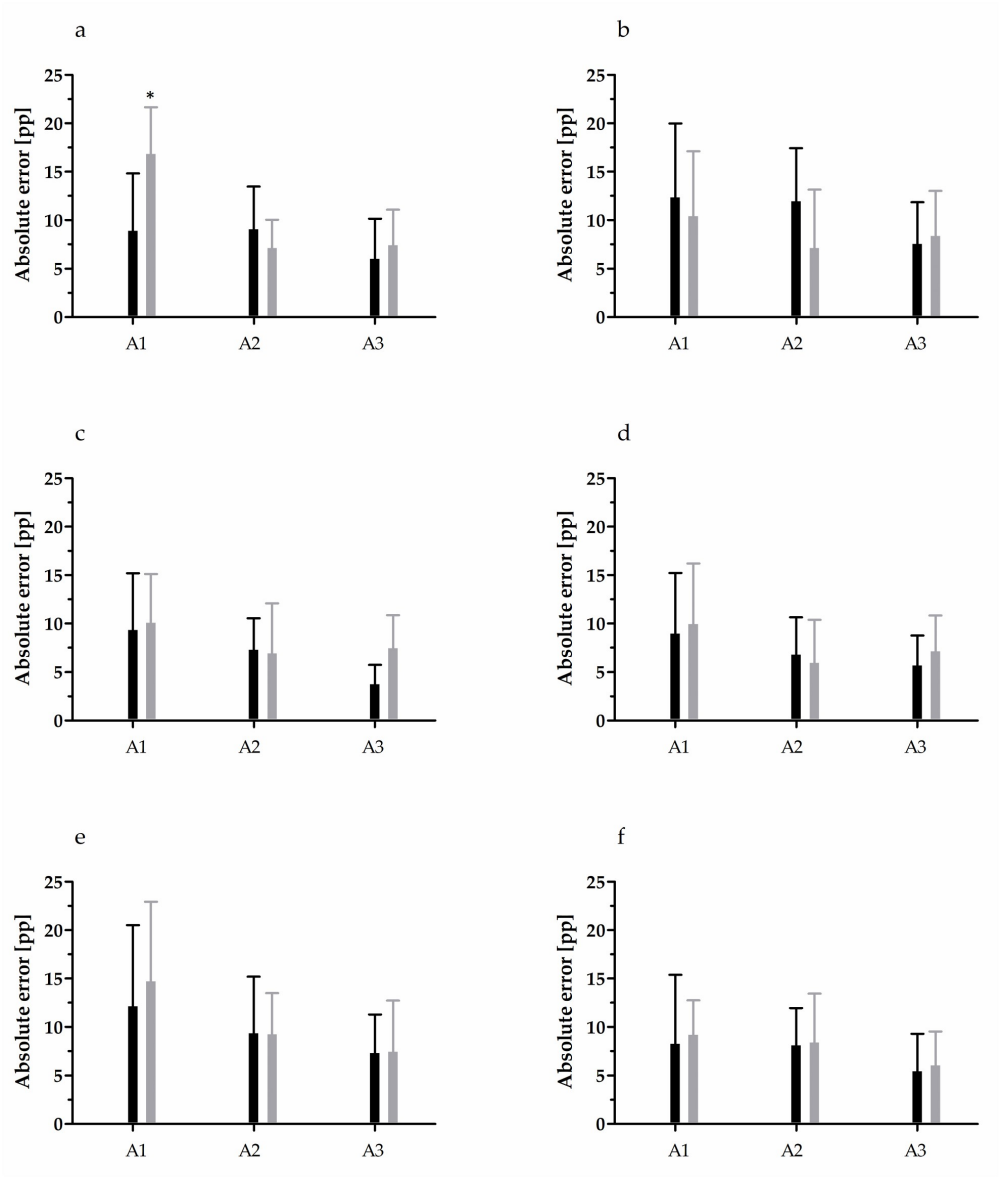
Absolute error in 50% maximal voluntary contraction force sense testing. Black bars, tennis players; grey bars, untrained controls. (a) Elbow extension; (b) elbow flexion; (c) radiocarpal extension; (d) radiocarpal flexion; (e) glenohumeral internal rotation; (f) glenohumeral external rotation. Data are presented as arithmetic mean ± standard deviation. Pp, percentage point; A1, 10–11-year-olds; A2, 12–13-year-olds; A3, 14–15-year-olds. * Significant difference vs. all other groups at p < 0.001.

The results of AE analysis in FS reproduction test are depicted in Figs 2 and 3. For the elbow joint, while no significant effect of the main factors on the AE of 20% MVC reproduction during elbow extension was detected, an interaction between the main factors was noted (F = 3.31, p = 0.04, η^2^ = 0.10) (Fig 2a). Further, the VE was significantly higher (F = 3.41, p = 0.05, η^2^ = 0.10) in 14–15-years-old tennis players than in younger peers (Table 3). For the AE of 50% MVC reproduction of elbow extension, a significant effect of both factors was noted; however, the interaction between them (F = 6.78, p < 0.01, η^2^ = 0.19) indicated that this was related to a very high error among the 10–11-years-old untrained controls in comparison to all other groups (Fig 3a). This error was caused by a considerably undershot target torque value, evidenced by the CE (Table 3). No effect was observed for elbow flexion.

**Table 3:** Constant (CE) and variable (VE) error in force testing (mean ± standard deviation).

Joint and activity		10–11-years-old		12–13-years-old		14–15-years-old	
		Tennis players (n = 12)	Untrained controls (n = 10)	Tennis players (n = 11)	Untrained controls (n = 11)	Tennis players (n = 12)	Untrained controls (n = 11)
20% maximal voluntary contraction (percentage points)							
**Elbow**	**CE**	1.63 ± 5.78	0.25 ± 3.42	1.15 ± 2.09	–0.10 ± 5.47	2.31 ± 3.89	1.65 ± 3.29
**extension**	**VE**	2.03 ± 1.46	1.66 ± 1.23	1.75 ± 1.59	3.20 ± 2.57	3.64 ± 2.54*	2.13 ± 1.35
**Elbow**	**CE**	1.22 ± 3.10	–0.58 ± 3.29	–0.68 ± 3.45	–0.20 ± 3.03	0.29 ± 2.82	–0.47 ± 2.90
**flexion**	**VE**	2.20 ± 2.35	1.81 ± 0.99	1.33 ± 0.88	0.86 ± 0.62	1.61 ± 1.26	1.08 ± 0.98
**Radiocarpal**	**CE**	1.61 ± 5.28	0.34 ± 2.68	2.31 ± 2.35	2.43 ± 2.18	4.16 ± 4.36	3.05 ± 3.81
**extension**	**VE**	2.77 ± 2.75	2.59 ± 2.39	1.65 ± 2.02	1.91 ± 1.39	1.92 ± 1.48	2.13 ± 2.29
**Radiocarpal**	**CE**	–1.77 ± 3.89	–2.09 ± 2.87	–2.46 ± 2.37	0.15 ± 3.16	–0.83 ± 2.49	0.32 ± 5.02
**flexion**	**VE**	1.64 ± 1.59	2.23 ± 1.57	1.33 ± 1.22	1.81 ± 1.76	1.30 ± 0.96	2.14 ± 2.28
**GH**	**CE**	–1.45 ± 4.21	0.57 ± 3.66	–0.40 ± 2.18	3.02 ± 6.88	–0.52 ± 2.36	2.22 ± 5.38
**int. rotation**	**VE**	2.48 ± 2.78	1.76 ± 1.55	1.47 ± 1.83	3.72 ± 4.75	1.18 ± 1.21	2.07 ± 1.75
**GH**	**CE**	1.28 ± 3.12	–2.47 ± 1.97^&^	0.41 ± 3.14	2.23 ± 5.26	2.69 ± 3.56	0.39 ± 1.88
**ext. rotation**	**VE**	2.79 ± 2.68	1.96 ± 1.42	1.90 ± 0.74	3.12 ± 3.13	1.35 ± 1.89	1.13 ± 0.75
50% maximal voluntary contraction (percentage points)							
**Elbow**	**CE**	–2.40 ± 7.95	–14.24 ± 10.63^#^	–6.93 ± 5.84	–2.33 ± 5.19	–5.21 ± 4.43	–4.78 ± 4.81
**extension**	**VE**	6.99 ± 5.12	5.95 ± 3.73	5.04 ± 3.24	4.27 ± 3.93	4.17 ± 3.48	4.84 ± 3.06
**Elbow**	**CE**	–9.24 ± 11.40	–5.72 ± 11.16	–9.95 ± 6.66	–5.17 ± 7.56	–4.66 ± 7.36	–7.59 ± 5.19
**flexion**	**VE**	2.99 ± 1.76	3.18 ± 2.34	4.81 ± 5.51	3.08 ± 2.54	2.79 ± 2.05	4.54 ± 3.32
**Radiocarpal**	**CE**	–3.37 ± 8.77	–7.75 ± 6.24	–1.24 ± 7.30	–3.44 ± 7.57	–2.02 ± 3.69	–4.16 ± 6.32
**extension**	**VE**	5.47 ± 5.14	6.35 ± 5.34	3.50 ± 2.66	3.66 ± 3.25	2.14 ± 1.32	4.65 ± 2.82
**Radiocarpal**	**CE**	–6.18 ± 7.88	–9.31 ± 6.98	–2.27 ± 5.29	–1.00 ± 6.11	–3.93 ± 4.77	–4.50 ± 6.29
**flexion**	**VE**	4.65 ± 3.31	4.05 ± 3.06	5.24 ± 3.58	3.50 ± 3.79	3.22 ± 2.84	4.41 ± 2.10
**GH**	**CE**	–11.26 ± 9.28	–12.84 ± 9.25	–6.58 ± 9.11	–1.86 ± 9.93	–0.98 ± 7.97	–4.33 ± 8.18
**int. rotation**	**VE**	4.11 ± 3.21	5.28 ± 4.94	2.27 ± 1.52	4.26 ± 4.13	4.61 ± 2.53	2.71 ± 2.30
**GH**	**CE**	–5.17 ± 9.79	–6.18 ± 6.07	–4.27 ± 7.82	–5.78 ± 5.62	0.29 ± 6.55	–5.35 ± 4.37
**ext. rotation**	**VE**	2.38 ± 2.09	3.74 ± 3.26	3.46 ± 2.41	4.95 ± 4.89	2.72 ± 2.52	3.08± 1.75

GH, glenohumeral joint; ext., external; int., internal.

* Significant difference vs. 10–11-year-old untrained controls and tennis players, and 12–13-year-old tennis players;

^#^ significant difference vs. all other groups;

^&^ significant difference vs. 12–13-year-old untrained controls, and 10–11- and 14–15-year-old tennis players at p < 0.05.

Considering the radiocarpal joint, only the reproduction of 50% MVC showed a significant effect of age. The AE of radiocarpal extension of the participating 10–11-year-olds, regardless of the training status, was 70% higher (F = 4.66, p = 0.01, η^2^ = 0.14) than that of 14–15-year-olds. For radiocarpal flexion, the youngest groups showed a similar tendency (F = 3.02, p = 0.06, η^2^ = 0.09), with the AE 46.5% higher than that of the other age groups. This was caused by a significantly undershot target torque (F = 5.09, p <0.01, η^2^ = 0.15), as evidenced by the CE (Table 3).

The most diverse readings were obtained for the glenohumeral joint. In the case of 20% MVC reproduction of the internal rotation, overall, the AE of tennis players was significantly lower (by 40.6%; F = 9.63, p < 0.01, η^2^ = 0.14) than that of untrained peers; the CE was also lower (F = 5.61, p = 0.02, η^2^ = 0.09, Table 3). However, as indicated by the interaction of the two factors (F = 5.31, p = 0.01, η^2^ = 0.16), this was only because of the difference in 12–13- and 14–15-year-old untrained controls (Fig 2e). In the case of 50% MVC reproduction of the internal rotation, a significant effect of age was observed for both, the AE (F = 5.29, p = 0.01, η^2^ = 0.15) and CE (F = 6.96, p < 0.01, η^2^ = 0.19). The AE of the 10–11-year-olds, regardless of the training status, was up to 81.5% higher than that of older age groups. This was associated with an undershot target torque value (over 4.4 times) in comparison with the older age groups. Lastly, a significant interaction was observed for the AE (F = 4.25, p = 0.02, η^2^ = 0.13) and CE (F = 3.86, p = 0.03, η^2^ = 0.12) of the glenohumeral external rotation during 20% MVC reproduction (Fig 2f and Table 3).

## Discussion

The first aim of the current study was to follow the level of proprioception in the upper limb by evaluating the differences in JPS and FS in different upper-limb joints in three consecutive age groups, namely, 10–11-, 12–13-, and 14–15-year-old girls. To the authors’ knowledge, this is the first study to show differences in the FS reproduction task in consecutive age groups in three different upper-limb joints. It is worth noting that the AE decreased with age only for the more demanding task, i.e. 50% MVC reproduction. Furthermore, the effect of age was only observed for each specific muscle function in the tested joints, not for the opposite function. The specificity of muscle function performance could be associated with the particular role of the investigated muscle groups. The difference concerned radiocarpal and elbow extensors and glenohumeral internal rotation muscles, which are mostly involved in phasic activity. Therefore, the observed difference could be associated with a higher content of fast-twitch muscle fibres and a lower capability to recruit type II motor-units [23] (especially during more demanding tasks) of the extensor muscles in adolescents.

The developmental difference in the magnitude of error in JPS was only observed for the elbow joint, between 10–11-year-olds and 12–15-year-olds. Similar outcome was observed in a study of young gymnasts and untrained peers, in which, regardless of sport training, the AE for the elbow joint of 9–11-year-old boys during both active and passive reproduction was two times higher than that of adults [24]. The lack of differences in the accuracy of glenohumeral joint positioning could be associated with the observation that motor coordination of proximal body parts develops faster than that of the distal body parts [25]. However, this does not explain the lack of the effect of age on the radiocarpal joint. This could be explained by the overall lower range of motion in the radiocarpal joint, wherein the participants were reproducing joint position that was relatively closer to the end of range than was the case for the other joints. It was previously shown that the higher firing from muscle spindles close to the end of range of motion leads to better JPS performance [26]. Furthermore, the contribution of motor commands to JPS in the glenohumeral and elbow joints is different [27] and, thus, probably also the rate of development, which was not observed in the current study.

The second aim of the study was to determine the differences between young tennis players with varying amounts of tennis experience and their untrained peers in the proprioception modalities. Athletes with long-term tennis practice are characterized by adaptation in proprioception, which is related to the specific demands of tennis training and competition [17, 18]. It was previously shown that the hip and knee JPS performance is better in more experienced tennis players than that in novice players [20]. In the current study, the effect was observed only in the glenohumeral joint, wherein all tennis players performed better than their untrained counterparts. The differences in the particular joints could stem from the specifics of tennis training, where the glenohumeral joint plays a dominant role in swinging the tennis racquet. It was previously shown that the intra-muscle coordination in the upper limb is initialized by the position of the most proximal joint, with the remaining distal joints adjusting to the first one [28]. Most likely, this explains why the differences between tennis players and untrained peers in JPS performance were most prominent in the glenohumeral joint in the current study. Similarly, in a previous study including young male artistic gymnasts, no effect of sport training on the elbow joint position acuity was noted for 9–11-year-olds and adults [24]. It should be pointed out that no other joint was evaluated in the gymnasts in the cited study.

In the current study, we showed that the FS difference between tennis players and their untrained peers was very varied and related to the specific functions and joints. While athletes with long-term tennis practice exhibit sport-related adaptations in the proprioception, also a joint specificity of upper limbs proprioception perception is observed in individuals regardless of sports training [27, 29, 30]. Therefore, the observed difference between tennis players and untrained peers in FS performance is a result of both the training- and joint-related factors. Until now, no study has evaluated FS in three joints of the upper limb in athletes and untrained individuals before. Below, we present an attempt to explain some differences in particular joints.

While we observed differences for the elbow and glenohumeral joints, no differences were noted for the radiocarpal joint. For the elbow joint, differences between tennis players and untrained peers were observed only in the extension function in both, the less and more demanding reproduction tasks, i.e. 20% and 50% MVC, respectively. In previous studies, the triceps brachii muscle of the dominant limb of tennis players showed up to 25% hypertrophy in comparison with that in the contralateral limb in adults [31] but not in prepubescents [32]. It was also shown that in tennis players, the elbow extensor (triceps brachii) is activated before impact to provide greater stiffness of the muscle—tendon complex to stabilize the elbow and glenohumeral joint before the impact, and also to anticipate the racquet slowdown during the follow-through phase in forehand stroke [33, 34]. This indicates that tennis players use elbow extensors for elbow coordination to a greater extent than their untrained peers in daily life, where elbow flexors are predominantly used. Most likely, that is why the differences between tennis players and untrained peers in FS were visible only for the extension function of the elbow joint in the current study. It was previously shown that other activities, such as gymnastic training, can increase the FS reproduction in both, the extension and flexion of the elbow joint, but only in the 50% MVC task [24]. This suggests that specific training can improve the FS performance as per the demands of the particular sport.

On the other hand, in the current study, the differences between tennis players and untrained peers in the glenohumeral joint was observed for both, internal and external rotation, but only in the 20% MVC reproduction task. Tennis players exhibited better glenohumeral internal rotation performance than untrained controls because of their greater utilization of this movement during training and competition [35]. By contrast, the external rotation error of the oldest tennis players evaluated was higher than that of untrained peers. It is difficult to compare the study outcome with those of other studies because of limited research into FS in tennis players. According to previous studies, in tennis players, the muscle strength ratio of the internal/external rotators is higher [36], with a decreased range of motion in internal rotation [37, 38]. We made similar observations in the current study, with the ratio values for tennis players, which increased with the tennis-playing experience, higher than those for untrained peers (data not shown). This might explain why the performance of the oldest group of tennis players was worse than that of untrained peers during 20% MVC reproduction for the external rotators. Such kind of proprioception insufficiencies may increase the risk of injuries [13, 39, 40], and it should be investigated more in this context.

The main limitation of the current study is that the particular target angle in the JPS evaluation is represented by an absolute angular value instead of a value relative to the individual’s range of motion. While there is evidence for more accurate performance near the end of range of motion [26], in the current study, the chosen joint angle values were generally under 50% of the normative range of motion. Hence, this should not have impacted the current JPS evaluation [41–43]. As another limitation of the study, it could be argued that the observed differences are related more to the specific selection of children with an initial specific sport predispositions instead of tennis training alone, since this is a cross-sectional study. Some effect of the predisposition cannot be excluded; however, we clearly showed here that tennis players from the same sport club and with a similar training regime but a different age and training experience performed differently from each other, as well as from their untrained counterparts. Another issue is that only the female players have participated in the study. It was purposely done to maintain similar training approach and methods in consecutive age groups as the same coaching team was involved in each group. This could not be achieved in particular sport club if the boys would be also included in current study.

## Conclusion

The developmental differences in FS in the upper limb varied and were related to the specific functions and joints. On the other hand, the elbow joint was the only joint that exhibited developmental differences in JPS performance. This implies that the upper limbs level of proprioception development in 10–15-year-old girls differs depending on the proprioception modality, evaluated joint, and function. Therefore, studies investigating training effects on upper limb proprioception in young girls should consider various forms of proprioception assessment and should not be limited to one joint only.

The 10–13-year-old tennis players showed elbow extensor FS performance at the level of the older participants. The 14–15-year-old tennis players were characterized by superior FS internal rotation performance in the glenohumeral joint at the expense of its external rotation. Considering JPS performance, in the 10–15-year-old girls, tennis practice-related differences were only observed in the glenohumeral joint. This suggests that the proprioception of glenohumeral joint internal rotation and elbow extension can play an important role in tennis player development. Further studies investigating the relationship of proprioception performance and tennis skills level should be undertaken to explore the significance of the study findings in the context of a sports career in tennis.

## Supporting information

S1 File Raw data set. (XLSX)
